# Effect of Shirts with 42% Celliant™ Fiber on tcPO_2_ Levels and Grip Strength in Healthy Subjects: A Placebo-controlled Clinical Trial

**Published:** 2019

**Authors:** Ian L Gordon, Seth Casden, Mark Vangel, Michael R Hamblin

**Affiliations:** 1 Veterans Affairs Long Beach Healthcare System 5901 E, 7th Street, Long Beach, California, USA; 2 Hologenix LLC, Santa Monica, CA 90403, USA; 3 Department of Biostatistics, Massachusetts General Hospital, Boston, MA 02114, USA; 4 Wellman Center for Photomedicine, Massachusetts General Hospital, Boston, MA 02114, USA; 5 Department of Dermatology, Harvard Medical School, Boston, MA 02115, USA

**Keywords:** Far-infrared emitting fabric, Transcutaneous oxygen, Grip strength, Blood pressure, Clinical trial

## Abstract

Celliant™ fabric contains quartz, silicon oxide and titanium oxide particles embedded into polymer fibers. Garments woven with Celliant™ yarns can be activated by body heat (conduction, convection and radiation) and remit the energy as far infrared radiation (FIR) back into the body. Wearing Celliant garments has been shown to increase blood flow and oxygen levels in the skin. In the present study we recruited twenty-four healthy volunteers (18–60 years of age) to wear a placebo shirt for 90 minutes, and after a 15-minute break, to wear a real Celliant shirt for 90 minutes. The mean transcutaneous oxygen (tcPO_2_) measured over two sites (biceps and abdomen) was significantly increased at 3 time points (30, 60, and 90 minutes) by between 5–8% (P<0.05) in Celliant vs. placebo. The mean grip strength in the dominant hand measured at 90 minutes was 12.44% higher after wearing Celliant vs. after placebo (p=0.0002). There was a small but significant increase in systolic blood pressure (113.71 vs. 109.38; p=0.02) but no statistically significant changes in diastolic or mean blood pressure, heart rate, or skin temperature. These data provide more evidence of the physiological effects of FIR emitting garments and suggest they could be used for athletic training and recovery.

## Introduction

Far infrared emitting fabrics and garments are becoming increasingly popular both for general health and wellness, and for the treatment of various medical conditions. Far-infrared radiation (FIR) therapy has long been used for the treatment of a wide range of diseases and conditions [1–3], including pain [4–6], wound healing [7], recovery from exercise [8], heart failure [9], and disturbed sleep [10]. The FIR is often delivered by an electrically-powered device such as an infrared heat lamp, an infrared sauna, or a tourmaline and/or jade heating pad.

However, an alternative way to deliver FIR to the body employs clothing, bandages, or patches constructed from fibers embedded with ceramic particles that emit FIR when powered by the body heat of the wearer [3]. These fabrics consist of a variety of different mineral particles incorporated into the polymer fibers, which are then woven into garments or patches. The mechanism of action involves natural heat from the body (radiation, conduction and radiation) being absorbed by the fabric, which then re-emits the energy back into the body in the form of FIR as a broad peak with a wavelength centered about 10 μm according to the Stefan-Boltzmann law.

The effective power density emitted by these fabrics is low (<1 mW/cm^2^) compared to that emitted by externally-powered devices (>10 mW/cm^2^). Nevertheless, garments, bed sheets, bandages, and patches can be worn for much longer times, many hours or even continuously, allowing a physiologically relevant amount of FIR to be delivered to the body. At a molecular level the mechanism is thought to involve the absorption of the FIR by “nanostructured water” which forms at hydrophobic/hydrophilic interfaces at cellular membranes including the mitochondrial membrane. The stimulation of mitochondria along with the release of nitric oxide, can increase cutaneous blood flow, tissue oxygenation, and produce beneficial effects such as reducing pain, inflammation, and stimulating wound healing and muscle recovery.

We previously showed [11] that the wearing of a Celliant shirt for 90 minutes could significantly increase the mean tcPO_2_ by 6.7% compared to a placebo shirt (p <0.0003) and also produce a small but significant increase in the arterial oxygen saturation (p=0.0002). Moreover these effects were independent of the sequence in which the two shirts were worn, thus eliminating a possible source of bias.

The present study was designed to confirm the previous findings of increased tcPO_2_ after wearing a Celliant shirt, and also to test the novel hypothesis that these physiological changes could result in improvement in an indicator of real-life muscular performance, namely the grip strength of the dominant hand.

## Material and Methods

**Figure 1:**
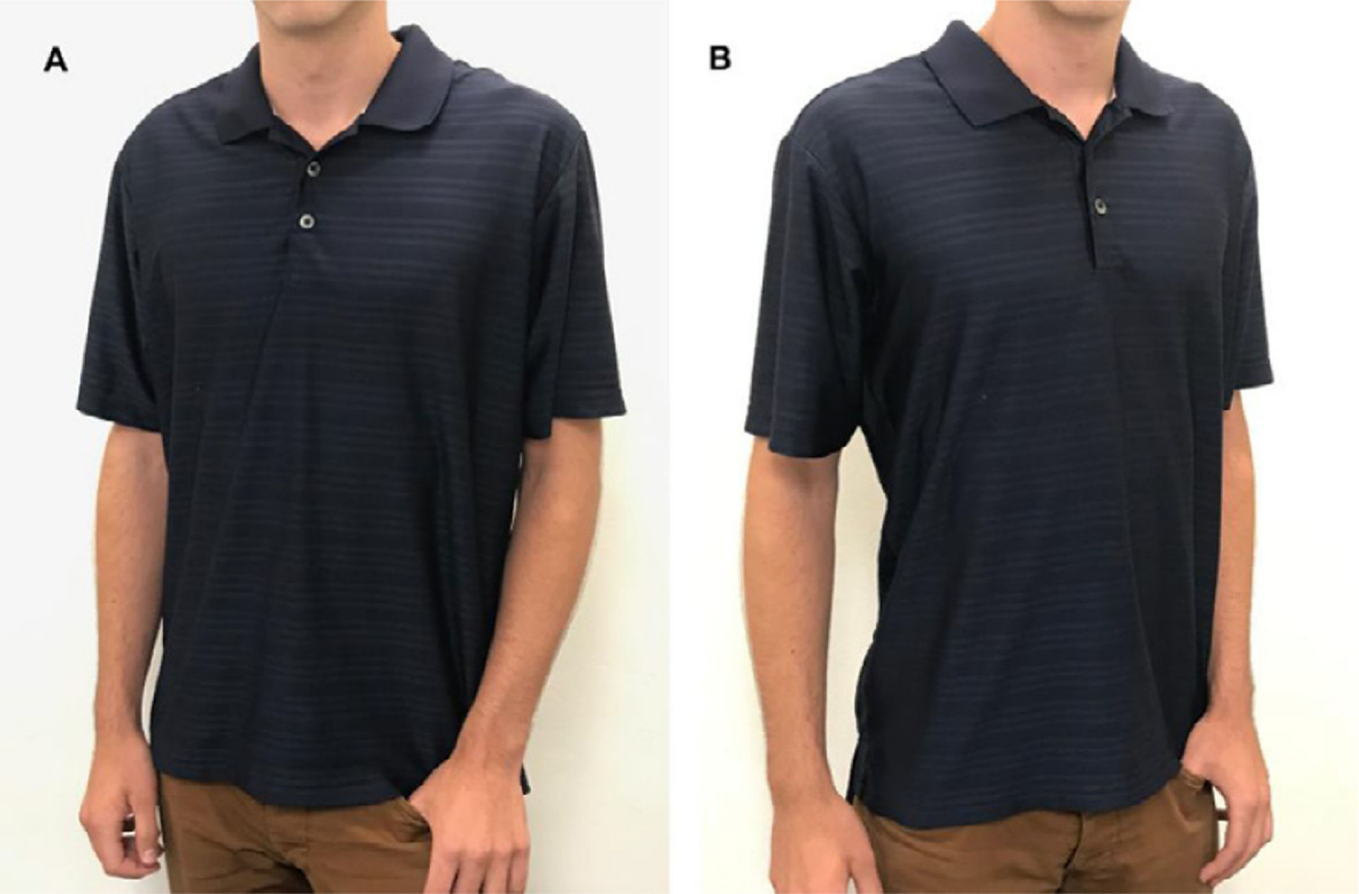
Photographs of golf-shirts containing (A) 42% Celliant (active) or (B) 0% Celliant (placebo).

Celliant™ technology is a patented process for adding micron sized optically active quartz, silicon oxide and titanium oxide particles to polyethylene terephalate (PET) fibers. The resulting Celliant™ yarns are woven into golf-shirts containing either 42% Celliant (active) or zero Celliant (placebo). The two shirts are shown in Figure 1.

Transcutaneous oxygen measurements were recorded using Radiometer TCM 30 Module supplied by Radiometer America, Inc. (Brea, CA), and modified Clarke Electrodes supplied by Radiometer America, Inc. Data was acquired using Perisoft Version 2.10 supplied by Perimed America, Inc. (North Royalton, OH). The grip strength was tested using the dominant hand using the Baseline Hydraulic Hand Dynamometer manufactured by Fabrication Enterprises Inc. (White Plains, NY).

### Clinical trial

Twenty-four healthy subjects (males and females between the ages of 18–60 years) were recruited under a protocol approved by the IRB via an on-line advertisement for subjects that paid $25. The trial was registered at clinicaltrials.gov NCT02798640 (https://clinicaltrials.gov/ct2/show/NCT02798640). The protocol was in accordance with the Declaration of Helsinki and verbal informed consent was obtained. Exclusion criteria included cardiovascular disease, smoking, pregnancy, recreational drug use within 6 months, or consumption of alcohol within 48 hours or caffeine within 3 hours of testing. The demographics of the subjects are given in Table 1.

### Procedures

The subjects underwent preparation as follows. The hair was shaved from the test sites (biceps of the dominant hand and center of abdominal wall); the skin surface was then abraded with a fine abrasive material; the stratum corneum was then removed by the use of lightweight adhesive tape; and finally, the probe site was wiped with an alcohol preparation swab.

Subjects were then situated in a seated position on a comfortable chair. The room temperature was maintained at a constant temperature over the duration of the study. Blood pressure, heart rate and body temperature were recorded at three intervals: before the test was administered, after the 90 minute placebo garment period and finally after the 90 minute Celliant™ garment period.

The transcutaneous oxygen electrodes were heated to 45°C and allowed to equilibrate on the skin for 10 minutes (until stable values were achieved). The resultant tcPO_2_ values were measured in mmHg.

Two self-adhesive fixation rings were affixed (biceps of dominant arm and abdomen) and the probes attached thereto. A buffer (KCl) solution was added (3 drops to each fixation ring). Each probe was calibrated to an assumed atmospheric pressure of 159 mmHg, 20.9% of the standard atmospheric pressure of 760 mmHg. Each subject therefore had the same baseline atmospheric pressure, thus compensating for any pressure changes due to weather variations during the periods of testing.

Measurements of tcPO_2_ were taken every two minutes during the 90-minute placebo and 90-minute active study periods. Blood pressure, heart rate and body temperature were recorded before and after each period. The entire study involved an acclimatization period (5 minutes) with vital sign measurement, placebo probe equilibration (10 minutes), placebo shirt period (90 minutes), break (15 minutes), active probe equilibration (10 minutes), active-shirt period (90 minutes). During the break (after the placebo period) subjects were encouraged to walk about, relieve themselves if necessary, and consume water and/or a small snack. After the break, the electrodes were reattached and equilibrated; subjects donned the second shirt and resumed quietly sitting.

The grip strength of the dominant hand was measured as follows. For each test of grip strength, the subject was seated with shoulder adducted and neutrally rotated, elbow flexed at 90°, forearm in neutral position, and wrist between 0° and 30° dorsiflexion and between 0° and 15° ulnar deviation.

During the preliminary acclimatization process, the subject was asked to squeeze the dynamometer initially at less than full strength simply to experience holding and using the device. The first measurement was taken after the 90-minute period of wearing the placebo garment. This served as the baseline or control measurement. The second measurement was taken following the 90-minute period after the Celliant™ garment was worn.

### Statistics

Grip strength was compared using a paired t-test. Time trends in tcPO_2_ were estimated using mixed-model regression with subject as a random effect. Data were smoothed as necessary using cubic regression splines. The data were analyzed using R [12].

## Results and Discussion

There was a small but significant increase in systolic blood pressure (113.71 vs. 109.38; p=0.02), but no statistically significant changes in diastolic or mean blood pressure, heart rate, or skin temperature as shown in Table 2. The mean transcutaneous oxygen (tcPO_2_) measured over two sites (biceps and abdomen) was significantly increased at 3 time points (30, 60, and 90 minutes) by between 6–10% (p <0.05) in Celliant vs. placebo as shown in Table 3. When the two different sites (biceps and abdomen) were compared, it could be seen that the increase in the tcPO_2_ measured at the biceps site was higher than that measured for the abdomen site and the corresponding p values were lower. This is probably because the biceps site was covered by the shirt while the abdomen was not.

**Figure 2:**
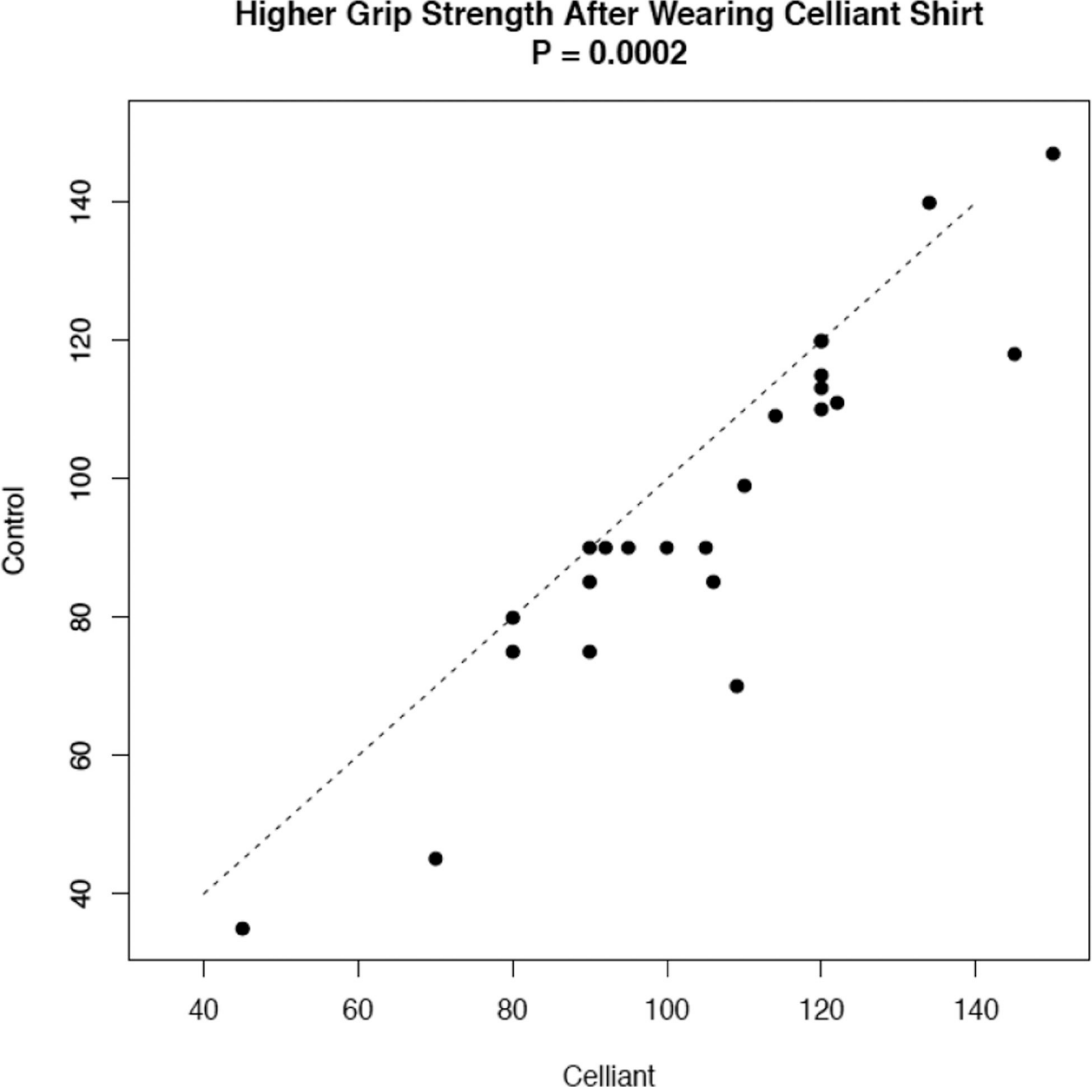
Plot of grip strength measured in 19 subjects after wearing placebo shirt (control) and active shirt (Celliant). The diagonal line indicates equality; points below the line correspond to subjects who had higher grip strength after wearing the Celliant shirt. The P-value is from a paired t-test.

**Figure 3:**
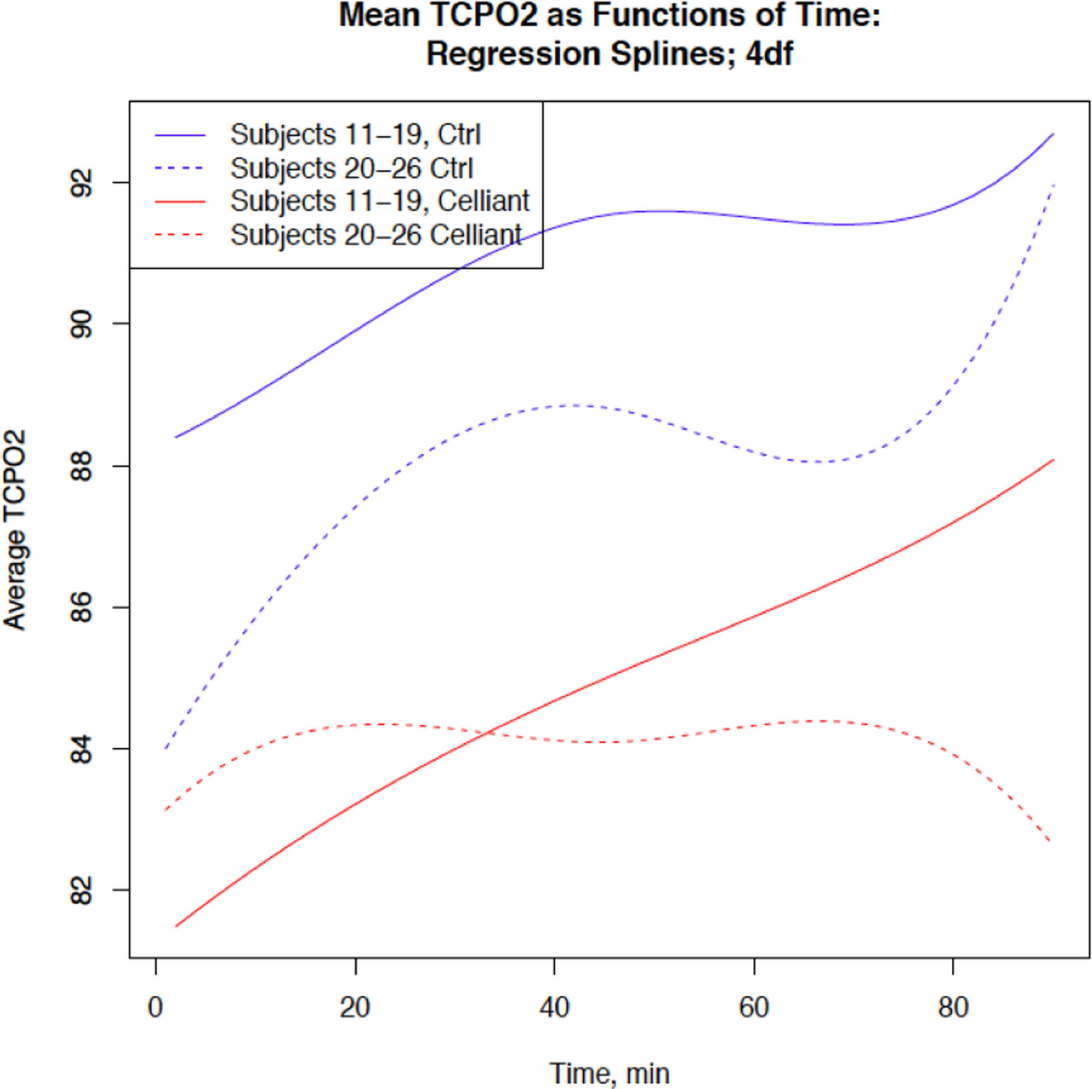
Average tcPO_2_ as functions of time for seven subjects with measurements at one-minute intervals (broken lines), and for eight subjects with measurements at two-minute intervals (solid lines), from a single probe location, for Celliant (red) and Placebo (blue) shirts. The values were first smoothed using cubic regression splines with four degrees of freedom and equally spaced knots, then averaged over subjects.

**Figure 4:**
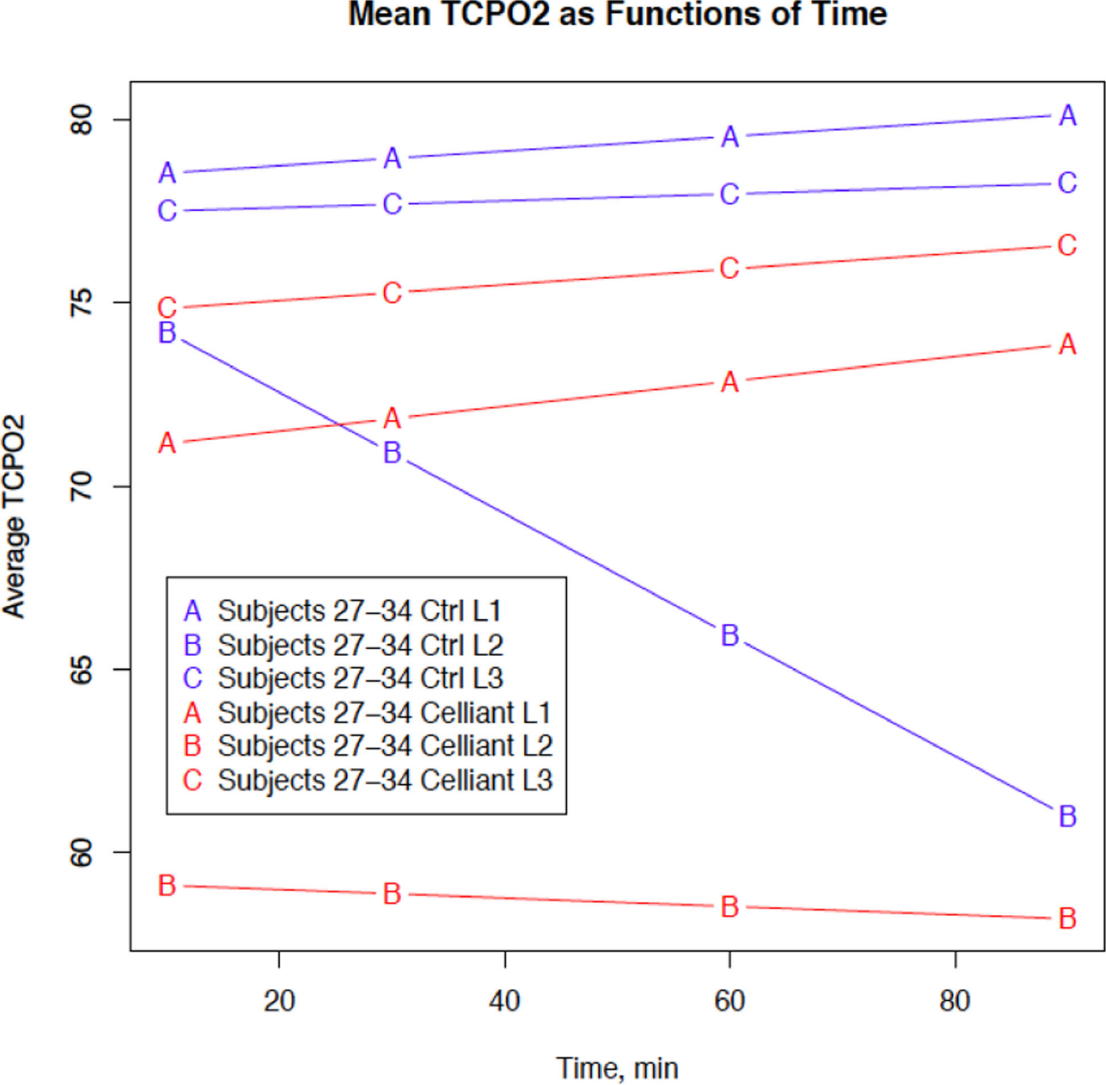
Average tcPO_2_ as functions of time for eight subjects with measurements at 10, 30, 60 and 90 minutes, from probes at three locations. Letters (A, B, C) and colors (red, blue) distinguish probe location and shirt, respectively.

The mean grip strength in the dominant hand measured at 90 minutes was 95.92 ± 26.36 after wearing the placebo shirt, and 105.20 ± 23.82 after wearing the Celliant shirt. This represents an increase of 12.44% (p=0.0002). There was an increase in grip strength for 19 subjects, no measurable difference in strength for four subjects, and a decrease for one subject as shown in Figure 2. For seven subjects with TCPO_2_ measurements at one-minute intervals, and also for a second group of eight subjects with measurements at two-minute intervals, the average values as functions of time are lower for Celliant shirts as shown in Figure 3. For eight subjects with TCPO_2_ measurements from four time points at each of three probe locations, the average values for the Celliant shirts are lower than the corresponding values for the Placebo shirt, for each location and measurement time as shown in Figure 4.

This study confirmed the previously reported increase in tcPO_2_ produced by wearing a Celliant shirt for 90 minutes [11]. However it also produced a somewhat surprising and impressive finding, namely that the grip strength in the dominant hand was significantly increased by wearing the Celliant shirt compared to the placebo shirt. There was a small increase in systolic blood pressure, but this is probably too small to represent any significant health hazard.

A previous study at Exponent Consulting compared the emissivity of PET fabric with or without Celliant particles using sophisticated optical spectroscopic techniques. The intensity of infrared emission between 7.5 to 14 μm was 2.1% higher when fabric containing 1.22% w/w Celliant, was compared to fabric without ceramic particles [12]. This finding was consistent with the finding that the absorption co-efficient of Celliant fibers in the infrared spectrum was higher than the absorption co-efficient of pure PET fibers. In other words, the PET fibers were semi-transparent to infrared radiation, while the Celliant particles were opaque. A follow-up study from the same group [13] examined in more detail the influence of Celliant particles on the infrared reflectance of the PET fabric, and measured the transmission, and absorption. The findings confirmed that the addition of ceramic particles to PET fabric led to increased incidence of infrared radiation upon the skin at wavelengths longer than 4 μm, with a maximum effect at approximately 9.4 μm. The effect was attributed to increased absorption of infrared radiation at shorter wavelengths and reemission at longer wavelengths.

The emission of FIR from ceramic particle-embedded fibers can interact with molecular and cellular structures by increasing the vibrational energy stored in chemical bonds, particularly in water clusters in cell membranes and cellular organelles. Pertubation of the vibrational energy of water clusters could affect the tertiary conformation of protein molecules tightly associated with this “nanostructured” water [14,15]. Low intensity FIR lamps and topically applied (non-powered) FIR-emitting ceramic materials have been shown to induce cellular changes *in vitro*, and produce physiologic changes in both preclinical animal models and clinical studies. In none of these studies were the effects shown to be associated with significant changes in temperature, consistent with the low power from both FIR lamps and non-powered ceramics in thermal equilibrium with skin (on the order of 0.1–1 mW/cm^2^) [3].

Ting-Kai Leung and colleagues in Taiwan have studied the effect of FIR-emitting ceramic powders in a range of non-clinical biological studies. In one report [16], they cultured murine myoblast cells (C_2_C_12_) with bags of ceramic powder placed under the culture plates and found that FIR irradiation improved cell viability and prevented lactate dehydrogenase release when hydrogen peroxide was added, and also increased the intracellular levels of nitric oxide and calmodulin. They also employed electro-stimulation of amphibian skeletal muscle, and found that FIR emitting ceramics delayed the onset of fatigue, induced by muscle contraction. They went on to show [13] that ceramic-emitted FIR (cFIR) could increase the generation of intracellular nitric oxide in breast cancer cells and inhibit growth of murine melanoma cells. Similarly, they found [14] that cFIR increased calmodulin and nitric oxide production in RAW 264.7 macrophages. cFIR also increased the viability of murine macrophages with different concentrations of H_2_0_2_. In the same study, it was also shown that cFlR blocked ROS-mediated cytotoxicity (shown by measurements of cytochrome c and the ratio of NADP+/NADPH). The Leung group went on to study [15] a rabbit model of rheumatoid arthritis in which rabbits received intra-articular injections of lipopolysaccharide (LPS) to induce inflammation that mimics rheumatoid arthritis. FDG-PET scans were used to monitor the inflammation 16 hours and 7 days after the LPS injection. Rabbits were treated with cFIR in a cage surrounded by paper sheets impregnated with a thin layer of the ceramic powder, while the control group were surrounded by the same sheets without the material. Comparison of the final and initial uptakes of FDG in the LPS-injected left knee-joints of the rabbits indicated decreases in the cFlR exposed group compared to the control group indicating that FIR reduced inflammation.

With regard to clinical studies, FIR-emitting ceramics and fabrics have been employed both as ceramic discs held next to the body, and as garments or patches manufactured from FIR emitting ceramic material and subsequently applied to the human body.

For instance, a blanket containing discs was reported to improve quality of sleep [2]. 542 users of far-infrared emitting disks embedded in bedclothes revealed that the majority of the users reported a subjective improvement in their health, by completing a questionnaire. These improvements included the disappearance or reduction of feeling cold, stiffness of muscles in the shoulders, loins and legs, and improved sleep.

Gloves constructed from FIR emitting fabrics have been reported to treat arthritis of the hands and Raynaud’s syndrome [16]. This was a randomised placebo controlled study in 60 patients who suffered from Raynard’s syndrome, and reported significant improvements in subjective measures of pain and discomfort and in objective measures of temperature, grip and dexterity. Another study looked at pain reduction in fibromyalgia using a bioceramic shirt [17]. The study recruited 39 female patients (20 active and 19 placebo) who wore the shirts 8 hours/day for 60 days. The women in the active group showed a significant reduction in pain in the VAS (p<0.001), fewer tender points (p<0.001), improvement in the algometer score (p<0.001), and a significant reduction in FM symptoms and daily tablet intake (p<0.001). No significant changes in the placebo group were found.

A study employed socks made from PET fibers incorporating Celliant particles, designed to treat chronic foot pain resulting from diabetic neuropathy or other foot disorders [18]. A double-blind, randomized trial recruited 55 subjects (38 men, 17 women, average age 59.7 ± 11.9 years), 26 with diabetic neuropathy and 29 with other pain etiologies. Subjects were provided 3 pairs of socks (Celliant or placebo) in a closed container, and asked to wear them exclusively for the next two weeks. One and two weeks after the end of the period they filled out the same panel of questions. Greater reduction in pain was reported by Celliant subjects for 8 of the 9 pain questions employed, with a significant (p=0.043) difference between controls and Celliant for McGill question III. In neuropathic subjects, Celliant caused greater pain reduction in 6 of the 9 questions, but not significantly. In non-neuropathic subjects 8 of 9 questions showed greater pain reduction with the Celliant socks.

The increase in tcPO_2_ observed in the present study is likely to be a consequence of increased oxygen availability in the tissue receiving FIR, possibly through a vasodilatory effect in the dermal circulation or, alternatively, effects on oxygen binding to hemoglobin. The increase in grip strength after only 90 minutes of wearing the shirt, is probably also due to increased oxygen availability and improved blood flow to the muscles of the dominant hand. Although our understanding of the mechanisms responsible for the effect of ceramic polyester composites on human physiology is still incomplete, our data confirm that it is a real scientific phenomenon. Even without completely understanding the effect, it may be possible to design ceramic polyester composite garments that can improve strength and performance in athletic training, and improve muscle recovery after exercise. The recent decision by the US FDA that Celliant garments will be regulated as medical devices and as general wellness products (http://www.medicaldevices-business-review.com/news/fda-determines-celliant-products-meet-criteria-as-medical-devices-260717-5882229) encourages clinical testing in multiple disease indications.

## Conclusion

The data from this study suggests that wearing Celliant™ fabric shirts for only 90 minutes can increase tissue oxygenation in the skin, and increase grip strength in the dominant hand.

## Figures and Tables

**Table 1:** Demographics of subjects (mean ± SD).

Total subjects	24
Males	17
Females	8
Age (years)	30.05 + 10.56
Weight (lbs)	167.2 ± 42.9
Body mass index (BMI)	25.7 ± 5.5

**Table 2:** Physiological variables (mean ± SD).

	Placebo	Celliant	P value
Systolic BP mmHg	109.38 ± 15.74	113.71 ± 14.0	0.0198
Diastolic BP mmHg	70.96 ± 9.05	71.38 ± 10.58	0.7177
Mean BP mmHg	83.76 ± 10.64	85.49 ± 11.39	0.1207
Pulse Rate	65.67 ± 10.17	66.08 ± 10.85	0.8127
Skin Temperature °F	92.63 ± 1.95	92.75 ± 2.21	0.6893

**Table 3:** Mean tcPO_2_ data recorded at the biceps and abdominal sites (mean ± SD).

	Placebo	Celliant	% increase	P value
Mean 30 min	75.4 ± 19.37	79.63 ± 14.0	5.61	0.0417
Mean 60 min	74.81 ± 19.52	79.52 ± 19.53	6.29	0.0196
Mean 90 min	76.08 ± 20.05	81.62 ± 20.27	7.28	0.0029
